# Towards mainstreaming of biodiversity data publishing: recommendations of the GBIF Data Publishing Framework Task Group

**DOI:** 10.1186/1471-2105-12-S15-S1

**Published:** 2011-12-15

**Authors:** Tom Moritz, S Krishnan, Dave Roberts, Peter Ingwersen, Donat Agosti, Lyubomir Penev, Matthew Cockerill, Vishwas Chavan

**Affiliations:** 1 1968½ South Shenandoah Street, Los Angeles, California 90034-1208, USA; 2 Aundh, Pune 411007, India; 3 Zoology Microbiology Research Group, Zoology Department, Natural History Museum, Cromwell Road, London SW7 5BD, UK; 4 Royal School of Library and Information Science, Birketinget 6, Copenhagen, DK 2300, Denmark; 5 Oslo University College, Pb 4 St Olavs Plass, 0130 Oslo, Norway; 6 Plazi, Zinggst. 16, 3600 Bern, Switzerland and American Museum of Natural History, Central Park West at 79th Street, New York NY 10024, USA; 7 Institute of Biodiversity and Ecosystem Research, Bulgarian Academy of Sciences and Pensoft Publishers, 13a Geomilev Street, 1111 Sophia, Bulgaria; 8 BioMedCentral Ltd, Floor 6, 236 Gray's Inn Road, London WC1X 8HB, UK; 9 Global Biodiversity Information Facility Secretariat, Universitetsparken 15, DK 2100, Copenhagen, Denmark

## Abstract

**Background:**

Data are the evidentiary basis for scientific hypotheses, analyses and publication, for policy formation and for decision-making. They are essential to the evaluation and testing of results by peer scientists both present and future. There is broad consensus in the scientific and conservation communities that data should be freely, openly available in a sustained, persistent and secure way, and thus standards for 'free' and 'open' access to data have become well developed in recent years. The question of effective access to data remains highly problematic.

**Discussion:**

Specifically with respect to scientific publishing, the ability to critically evaluate a published scientific hypothesis or scientific report is contingent on the examination, analysis, evaluation - and if feasible - on the re-generation of data on which conclusions are based. It is not coincidental that in the recent 'climategate' controversies, the quality and integrity of data and their analytical treatment were central to the debate. There is recent evidence that even when scientific data are requested for evaluation they may not be available. The history of dissemination of scientific results has been marked by paradigm shifts driven by the emergence of new technologies. In recent decades, the advance of computer-based technology linked to global communications networks has created the potential for broader and more consistent dissemination of scientific information and data. Yet, in this digital era, scientists and conservationists, organizations and institutions have often been slow to make data available. Community studies suggest that the withholding of data can be attributed to a lack of awareness, to a lack of technical capacity, to concerns that data should be withheld for reasons of perceived personal or organizational self interest, or to lack of adequate mechanisms for attribution.

**Conclusions:**

There is a clear need for institutionalization of a 'data publishing framework' that can address sociocultural, technical-infrastructural, policy, political and legal constraints, as well as addressing issues of sustainability and financial support. To address these aspects of a data publishing framework - a systematic, standard approach to the formal definition and public disclosure of data - in the context of biodiversity data, the Global Biodiversity Information Facility (GBIF, the single inter-governmental body most clearly mandated to undertake such an effort) convened a Data Publishing Framework Task Group. We conceive this data publishing framework as an environment conducive to ensure free and open access to world's biodiversity data. Here, we present the recommendations of that Task Group, which are intended to encourage free and open access to the worlds' biodiversity data.

## Background

### Data: usage and definitions

The term 'data' [1,2] has two primary uses. One, specific to the information technology community, refers to any machine readable code that allows information to be read by, stored in, accessed by or shared by computers. For example, the United States National Science Foundation 'DataNet' program defines data as: "Any information that can be stored in digital form and accessed electronically, including, but not limited to, numeric data, text, publications, sensor streams, video, audio, algorithms, software, models and simulations, images, etc." [3]*.* Under this definition, theoretically everything can be 'digitized' and become 'data'. This 'bits and bytes' definition of data challenges us to ask what cannot - if digitally captured - be considered data?

A second usage refers to data in a more precise, epistemic way as: "Precise, well-defined representations of observations, descriptions or measurements of a referent (object, phenomena or event) recorded in some standard, well-specified way" [4].

In this report, we use this latter definition, although stipulating that such data may be technically formatted as text (descriptions), as maps, as visual images or audio recordings, as signals, as symbols or as numbers. This clarity is essential because in the context of biodiversity conservation in general, and a biodiversity data publishing framework in particular, data have a foundational place in the wisdom/knowledge hierarchy [5,6]. Information, knowledge and wisdom are synthesized from factual data, which are thus the basis for informed policies, decision-making and sustainable use of biotic resources. We urge that careful attention be consistently paid to which usage of the term 'data' is intended. Of elemental importance is that, to be useful, descriptions of data and of their provenance, lineage [7] and structure, normally collected as 'metadata', must exist.

### The volume of data

We are experiencing a tremendous increase in data generated by a variety of research processes. For example, a recent article, reviewing the growth of data in the International Nucleotide Sequence Database Collaboration (INSDC) notes: "the INSDC databases have grown to contain over 95 billion base pairs, reflecting an exponential growth rate in which the amount of stored data has doubled every 18 months" [8].

This increase has major implications for data management, data processing, data archiving and data accessibility. The potential for a tremendous signal to noise problem - challenging data users to effectively select relevant high quality data from a rapidly expanding corpus of data - suggests the urgent need, extensively and consistently, to implement well designed and deployed data management strategies. These strategies must carefully evaluate the relative returns on investment for incremental investments in data creation and collection [9]. It seems possible that only certain selected 'canonical' datasets of primary importance in guiding policy or in informing key decisions will be managed in full accordance with optimal recommendations. Determination of which datasets merit this level of optimal management seems best left to community mechanisms. However, recent challenges to the Intergovernmental Panel on Climate Change (referred to as 'climategate') make clear the importance of exhaustive documentation for datasets on which policies with major global, national and even local economic consequences are based [10]. In general, each researcher is responsible for the quality and integrity of their data; by direct release of data or by publication based on data, they are implicitly warranting that best professional practices have been followed in definition, creation and management of such data.

### Collections of data: databases, datasets and data tables

In colloquial scientific usage, collections of data are variously referred to as 'databases', 'datasets' and 'data tables', or merely as 'data'. In an effort to standardize usage for such collections, a recent publication [11] by several members of the Task Group has proposed a series of possible working definitions:

"'Data tables': represent precisely the set or sets of data upon which the analyses and conclusions of a given scientific paper are based. A data table is thus a discrete, fixed, time-bounded collection serving as a referent.

'Datasets': represent discrete collections of data underlying a scientific paper. Datasets are thus also fixed and time-bound though functioning in a more general way as a referent.

'Databases': represent larger, dynamic and more extensively coherent collections of data. By this definition, databases are not fixed or time-bounded but have properties of quality control and integrity and should provide the capacity for version control and version retrospection."

In the context of this article, we propose a clear distinction between fixed data tables that represent precisely the set or sets of data on which the specific analysis and conclusions of a scientific paper are based, datasets understood as a fixed and time-bound logical files presenting a collection of facts (observations, descriptions or measurements) formally structured into standard records, and dynamic databases representing larger and more extensive collections of data that may or may not include the precise data tables or more general datasets tables that are the referent(s) for a given scientific paper. Each data record is structured in fields with specifications for appropriate field content. In the context of this article, 'primary biodiversity data' is defined as digital text or multimedia data records providing facts about the instance of an organism: the what, where, when, how and by whom of the occurrence and the recording [12]. By this definition, data tables and datasets are inextricably linked to scientific papers and the publisher must assure consistent and secure access, in perpetuity, to referent data tables and datasets [11]. Thus, these collections of data impose the heaviest burden of responsibility on the publisher for sustained access.

With respect to the publishing of data, the customary practices of science suggest that data providing the evidence for conclusions drawn in a scientific paper or report should be available for review, evaluation and testing. This provision is fundamental to the objective practice of science as 'organized skepticism' [13]. Appropriate standards for testing data vary depending on the exact nature of the data. For example, *in situ* field data are evaluated by consideration of the field context, the methodology or apparatus used to collect data, the consistency or inconsistency with other comparable studies, the quality and detail of the reported observations, photographs or audio recordings, and material evidence (specimen, genetic sample, scat, tracks, and so on). The actual practicability of testing and assessing data is highly dependent on the thoroughness with which data are described and how completely the context for data collection is described. This leads logically to the question of metadata as a source of necessary contextual information about data.

### How data have meaning: metadata

'26.07' and '0.59998' are each an actual datum or 'data point'. It is immediately obvious that without any description of context for the creation and capture of data, an isolated datum is meaningless. Descriptive information is necessary to impart meaning. The former datum was recorded by Henry Cavendish in his "Experiments to Determine the Density of the Earth" (21 June 1798) and was published in the *Philosophical Transactions of the Royal Society of London *[14]. The Cavendish datum was a result of a humanly contrived experiment using a specially designed apparatus. The latter datum is a reading obtained from automated data loggers recording sap flow in Manzanita plants at the University of California James Reserve, Mt. San Jacinto, California (4 December 2007 11:37) and was recorded by a data logger in an as yet unpublished Microsoft Excel spreadsheet (Gary Geller, 2010 personal communication).

However, in the simple contexts disclosed above, we have learned that some agent conducted a data gathering exercise at a given date and time and at a described place. Inference of a probable general scientific domain or discipline for the data - for example, physics or ecology or botany - provides only a very general delimiter of the probable character of the data. We do not, for example, know the actual type of automated data logger used, its proper calibration, the actual details of its deployment in this instance of use, or the competence of the person using the data logger. Lacking this information and other information that would serve to validate the quality of the data presented, we are challenged with the need to develop and to provide more complete descriptions to make data fit for use and, in particular, fit for testing and evaluation.

### Provision of metadata

To avoid the risks of overly intricate and elaborate metadata standards that fail by requiring inordinate investments of time and resources, we suggest that metadata be initially designed to provide minimally adequate description for discovery and access to data. We propose that in the interests of optimal efficiency of effort, careful efforts be made to apply inference and recursion in creation of such minimally adequate metadata and that metadata subsequently be available for the continuing addition of fresh increments of metadata. This recommendation implies that metadata creation should be a continuous, collaborative process, not a single event. Specifically, with respect to museum collections, we recommend that links to relevant type specimens be included as a part of the metadata record.

Moreover, we believe that by careful application of qualified social tagging - that is, of indexing by expert users applying well-formed, ontologically suitable vocabularies and authority files - substantial development and enrichment of metadata records can be accomplished (this recommendation requires applications that can support a dynamic, coherent and iterative development of metadata over time) [15].

We also suggest that assessment of the fitness of metadata for use be considered from the 'demand side' by asking how data have typically been used to best effect in the creation of biodiversity knowledge and policy.

There are many technical publications - for example: Voss and Emmons 'Mammalian diversity in neotropical lowland rainforests: a preliminary assessment' [16], the US Fish and Wildlife Service's 'Statistical guide to data analysis of avian monitoring programs' [17] or Agosti *et al.*'s 'Ants: standard methods for measuring and monitoring biodiversity' [18] - that provide detailed descriptions of common data collection methods or of statistical processes applied to biodiversity data. Recently, the European Union Framework Projects 6 project EDIT (European Distributed Institute for Taxonomy) has developed a complete workflow, from data collection in the field to assembly of datasets and analyses [19,20]. These and many other works provide guidance in the development of standard ontologies for data description.

We recommend a research process that - from an ontological perspective - systematically reviews, analyzes and specifies how data can most efficiently be supplied to fit the needs of these primary biodiversity-monitoring processes. We suggest detailed survey and analysis of the primary and standard forms of processing that, by community consensus, are of greatest proven value and impact in biodiversity conservation. This assures that investments in data collection will have optimal probative force. Based in this analysis, standards can be 'reverse engineered' to produce data best suited to the demands of biodiversity conservation.

We also strongly recommend careful analysis of standards already under development. The Ecological Metadata Language (EML) [21] under continuing development has made significant progress, but we believe that the issues raised elsewhere in this report have yet to be addressed. Specifically, significant ontological work remains to be accomplished regarding the analysis and standard definition of biological field techniques, data transformation methods and statistical processes.

We also believe that the scripting capacity of standard statistical packages [22] and still emergent applications for documenting scientific workflow (such as Kepler [23]) may both have direct utility in recording the process and context for scientific data capture. A notable example of such workflow capture is in the Galaxy genomics platform [24]. Ontological research and development coupled with applications development should provide the necessary foundations for required descriptions of data.

In the social sciences, the Data Documentation Initiative, based at the University of Michigan's Interuniversity Consortium for Political and Social Research (ICPSR), has been underway for several years and is now at version 3.1 [25]*.* Similarly, a 2009 publication of the OECD has proposed a model template for metadata describing a published dataset [26]. The requirement of free text abstracts may provide an adequate frame for such detailed specification, but considerable additional work will be demanded, particularly in deriving minimal descriptive standards for discovery of biodiversity data.

The importance of metadata in exposing data to discovery becomes increasingly important as the units into which data are assembled become smaller. The molecular sequence repositories developed and maintained by International Nucleotide Sequence Database Collaborations (INSDC [27]), such as GenBank [28], ENA [29], and DDBJ [30], are perhaps among the best known example of a data repository, but although the search interfaces and the utility of data contained with GenBank are very limited (and especially geared for molecular biologists) its global prominence makes it an obvious search target. Biodiversity data in general are far more complicated and tend to be made available in smaller blocks, for example the data associated with a single publication. Locating and combining data relevant to a particular purpose thus becomes a goal in itself and is made possible through the existence of metadata using standard vocabularies.

### Open access and biodiversity data

Open access to primary biodiversity data is essential both for enabling effective decision making and for empowering stakeholders involved with and affected by the conservation of biodiversity [31-33]. Specifically with respect to scientific publishing, the ability to critically evaluate a published scientific hypothesis or scientific report is contingent on the examination, analysis, evaluation and, if feasible, re-generation of data on which conclusions are based. Biodiversity is not an exception to such data restrictions. For example, authors of a paper published on the failure of African game parks to successfully conserve large mammals were unable to present local data, gathered from reserve operators, who wanted it to be kept confidential [34].

There is broad emerging consensus in the scientific and conservation communities that data should be freely, openly available in a sustained, persistent and secure way [35-38]. However, many existing primary biodiversity data are neither accessible nor discoverable [39]. This issue is further compounded by lack of appropriate representation and/or visualization of available data and lack of linkability among distributed and heterogeneous data resources [40,41]. This adversely affects the optimal utility of the biodiversity data. Thus, an urgent need exists for the discovery of primary biodiversity data and its publication in the public domain.

For decades there have been declarations, statements, policies, and guidelines encouraging open access to primary scientific data [31,42]. With the establishment of the Global Biodiversity Information Facility (GBIF) in 2001, an attempt has been made to develop a global infrastructure to consolidate the discovery of the world's primary biodiversity data and to provide coherent access. Currently, the GBIF network facilitates access to nearly 304 million data records through its portal [43]. However, these primary biodiversity data records are just a fraction of the estimated volume of existing data [44-47]. This large volume of biodiversity data, collected by a vast number of biodiversity researchers and amateurs [31,47] remains largely undiscovered and unpublished. This is attributable, we believe, to a lack of encouragement, misperceptions of self-interest, or lack of infrastructural support. Although infrastructure support is increasingly available, the problem of appropriate professional recognition for institutions and individuals remains [31]. We believe that this lack of incentive remains a major impediment to the provision of free and open access to primary biodiversity data.

### The GBIF data publishing framework task group

The foregoing discussion emphasizes the need for a data publishing framework to evolve metrics and indicators that provides incentives to multiple actors involved in the generation of data. Recognizing the need for addressing social, policy, political, and technical issues influencing discovery and publishing through the GBIF network, the GBIF Data Publishing Framework Task Group (DPF TG) was commissioned in March 2009 [48]. The DPF TG was tasked with providing recommendations on (a) social, technical and policy interventions that would encourage publication of primary biodiversity data as a necessary and in-built step in the scientific data management cycle; (b) opportunities and mechanisms to incentivize and attribute credit for investment in primary biodiversity data publishing, from individual to institutional to national levels; and (c) mechanisms/processes for recognizing efforts of data publishers. The concept of the data publishing framework was described at the International Biodiversity Informatics Conference ('e-Biosphere 09') held in London in June 2009 [49]. In its meeting in June 2009, the DPF TG discussed issues influencing discovery and publishing of primary biodiversity data, and possible solutions in overcoming impediments.

### A data publishing framework for primary biodiversity data

During its meeting in June 2009, the DPF TG invested significant time in defining and determining the scope, and purpose of the data publishing framework for primary biodiversity data. The DPF TG recognized the need expressed by the data originators and information system/networks for data usage metrics and indicators to ensure that the overall utility and impact of their data management and publishing activities is objectively documented, leading to crediting of these activities as scientific activity on a par with the recognition received for conventional scholarly publication [31]. Furthermore, measures of scientists' productivity will be better informed through data publishing which requires a professional, cultural change in the recognition of scientific output [50]. Such an incentive mechanism would achieve increased data mobilization and increased recognition for data generation, both desirable outcomes for scientists.

Our discussion examined five primary components that comprise a data publishing framework. These components are (a) socio-cultural, (b) technical-infrastructural, (c) policy-political, (d) legal and (e) economic, and they support various activities of the data publishing cycle (see Figure 1 in [31]). These components are not only complementary, but are inter-dependent. Thus, there is no dependency on a sequence of components, as components need to be implemented concurrently. Therefore we define a data publishing framework as an environment conducive to ensuring free and open access to the world's primary biodiversity data. The core purpose of the framework is to overcome barriers or impediments affecting access to data and the publishing of data.

## Recommendations

On the basis of our understanding of issues influencing 'free and open access' discovery and publishing of the primary biodiversity data, to encourage institutionalization of the data publishing framework for discovery, publishing and use of primary biodiversity data, we make specific recommendations. The key words 'must', 'must not', 'required', 'shall', 'shall not', 'should', 'should not', 'recommended', 'may', and 'optional' in this document are to be interpreted as described in RFC 2119: 'Key words for use in RFCs to Indicate Requirement Levels' of the Internet Engineering Task Force [51].

Sharing of biodiversity data must be the expected norm. We stipulate that withholding of data - to protect precise localities for collectible or marketable plants or animals or for species of special concern - should be the exception and require explicit justification. We emphasize that such data represent a small fraction of biodiversity data and should not be allowed to dictate normal practice. We also stipulate that our call for access to biodiversity data does not supersede national or indigenous rights to regulate uses of biodiversity data as protection against commercial exploitation ('biopiracy'). To this end, we suggest close consultation and confirmation with CITES [52] and the TRAFFIC Secretariat [53] when questions of this kind occur. As a corollary, all contributors of data must receive appropriate, proportional recognition for their contributions of data. On this backdrop we offer 24 recommendations. Recommendation 1 is, however, the primary recommendation that leads to the other recommendations.

**Recommendation 1:** All data relevant to the understanding of biodiversity and to biodiversity conservation should be made freely, openly and effectively available.

**Recommendation 2:** GBIF must re-examine its current data resources endorsement model and scrutinize the current practice that national nodes or associate participant nodes are required to give endorsement before the data are discovered and indexed through GBIF network.

**Recommendation 3:** GBIF must engage mainstream scholarly publishers and scientific societies with scholarly publications to be part of the GBIF network, as a majority of them would qualify to be thematic/global/regional associate-participants.

**Recommendation 4:** GBIF must support the development of a tool to convert tabular data into resource description framework (RDF) formats conforming to a standard ontology. This would be highly desirable for small custodians/publishers but is primarily a tool for mainstream scholarly publishers. (Support for development of such an open source application should be sought from mainstream commercial publishers.) GBIF shall evaluate standards such as BioPax [54].

**Recommendation 5:** GBIF must facilitate discovery and mobilization of all streams/types of relevant biodiversity data. (This effort should - in close collaboration with others focusing on this development - include ontological analysis of the most important types of data to be considered, the elaboration of suitable working formats for that data, and the developing of mappings to/from such working formats to a standard RDF format for interchange purposes.)

**Recommendation 6:** GBIF should develop a set of supporting tools (such as templates) for biodiversity data to accommodate more than simple occurrence data. GBIF must increasingly engage with various biodiversity data communities.

**Recommendation 7:** GBIF must facilitate discovery of un-digitized and not yet published datasets together with indexing of published datasets (potentially to include semantic indexing based on RDF, to allow datasets to be filtered and retrieved with SPARQL queries). In this regard, we strongly endorse the recommendation by the GBIF Global Strategy and Action Plan for Mobilization of Natural History Collections data [55].

**Recommendation 8:** GBIF should review the use of legacy literature, such as is stored in Biodiversity Heritage Library (BHL), to explore uses of marked-up texts for data mining and capture of historical biodiversity information.

**Recommendation 9:** GBIF must explore and develop the capacity to run queries at the GBIF data portal to return harmonized, well formed XML and/or RDF such that fields can be extracted for subsequent analysis.

**Recommendation 10:** GBIF must expand and improve its metadata implementation framework to such that fitness for use of the data resource for intended use can be ascertained from metadata. For example, data records should identify lineage and provenance (where data originated, and from which data resource) of all contributed data - at least to the previous phase of data transformation. Further, we strongly encourage early implementation of the recommendations of the GBIF Metadata Implementation Framework Task Group [56].

**Recommendation 11:** GBIF must strengthen its network of mirror sites and distributed network of 'trusted digital repositories' (also called data hosting centers). In this regard we call on GBIF to ensure early implementation of the recommendations in this issue on data hosting infrastructure [57].

**Recommendation 12:** GBIF must explore the feasibility of using a cloud infrastructure to overcome barriers of investment and maintenance required for biodiversity data discovery and publishing, especially in the developing and under-developed regions of the world.

**Recommendation 13:** GBIF must ensure an early implementation of the recommendations of the GBIF Life Sciences Identifier (LSID)/globally unique identifier (GUID) Task Group [58]. We further emphasize the need for GBIF to adopt a stable and proven persistent identifier such as the 'digital object identifier (doi), rather than unstable persistent identifiers.

**Recommendation 14:** GBIF must explore the potential of the Data Usage Index (DUI) as potential incentivization mechanism to recognize efforts required for publishing of biodiversity data [31,59]. GBIF should develop a prototype of such an implementation.

**Recommendation 15:** GBIF must institutionalize a 'data citation mechanism' and establish a 'data citation service' facilitating deep-data citation, and registration and resolving of citations [26]. For the purposes of accountability and citation (attribution), all contributors of data to any aggregation should be identified and acknowledged. Individuals or institutions responsible for primary data have an obligation to make these ownership statements available to the aggregators, who are responsible for using them. The Dryad application, which uses DataCite to register dois, is an initial effort to address this concern [60]. In any data aggregation chain the aggregator at each level is responsible for identification of data sources from previous level of aggregation and its contributors. We believe that this provision avoids the complexity of comprehensive identity of all 'cascaded' data sources and contributors during the aggregation process. It is, of course, nevertheless the case that the validity and integrity of data are ultimately linked to the sum of the integrity and validity of all data processes in the lineage of data creation.

**Recommendation 16:** GBIF should investigate innovative mechanisms for discovery and publishing of primary biodiversity data in multiple languages. GBIF should commission a position paper detailing such mechanisms for potential uptake by the community.

**Recommendation 17:** GBIF must institutionalize the 'biodiversity informatics potential' (BIP) Index to demonstrate the potential and urgency for nations to implement biodiversity informatics [61]. In the long term GBIF must lead the periodic release of a 'global biodiversity information outlook' report analyzing the current state of biodiversity information to meet the local-to-global scale biodiversity targets.

**Recommendation 18:** GBIF must commission a strategy paper demystifying the concerns/issues related to intellectual property rights and primary biodiversity data. In this regard, the substantial work done by the Science Commons (for example the Science Commons Protocol for Implementing Open Access Data [62]) and the Open Knowledge Foundation [63] should have direct application.

**Recommendation 19:** GBIF should encourage sponsors of biodiversity research, whether government agencies, corporations or private foundations, to set mandatory requirements for free and open access to biodiversity data. GBIF should encourage that negotiations for overhead (indirect) cost contributions from funders should include calculations of cost for sustained digital infrastructure that is adequate for free and open sharing and the sustained, secure and persistent maintenance of data. Proposals should be expected to include adequate planning and financial provision for sustained data management and access. We further recommend that GBIF should encourage peer review processes that include rigorous scrutiny of past histories of successful sharing and should support the norm of state-of-the-art planning for sharing, not simply promises to "put data on the web".

**Recommendation 20:** GBIF must develop a plan to foster linkages between scholarly publishers and data publishers from the local to the global scale. GBIF should encourage that records of professional publication be evaluated - at least in part - on the basis of publication in open access journals that do not deny access through 'paywalls' and that provide support for sustainable open access to data.

**Recommendation 21:** GBIF should urge accreditation bodies for educational institutions and museums to require demonstrated evidence of capacity to support digital access and maintenance of data.

**Recommendation 22:** GBIF should encourage professional societies and professional disciplines to require evidence of effective sharing of data in evaluations for hiring, promotion and tenure.

**Recommendation 23:** GBIF should develop a conceptual 'landscape map' depicting GBIF's position, role, unique advantages and collaborative strategies, amid the many biodiversity and biodiversity informatics initiatives at local to global scales. This is very important given the broad reach of the earlier recommendations. It is important that the scope of the GBIF's own vision and mission is well defined, with a clear picture of how GBIF's role fits into a wider framework of sustainable development and of free and open access to biodiversity data.

**Recommendation 24:** GBIF must evaluate, prioritize and implement the recommendations made by its task groups - the Content Needs Assessment Task Group (CNA TG) [42], the Multimedia Resources Task Group (MRTG) [64,65], the Metadata Implementation Framework Task Group (MIFTG) [56], the LSID-GUID Task Group (LGTG) [58], the Observational Data Task Group (ODTG) [66] - and in the Global Strategy and Action Plan for Natural History Collections Data (GSAP-NHC) [55] and recommendations on e-learning recommendations [67], Knowledge Organization System (KOS) [68], and fitness for use [69].

## Discussion

These recommendations grew out of our discussion in June 2009. Since then, there have been subsequent revisions and modifications of the recommendations and some additions. Chavan and Ingwersen [31] further elaborated on various components of the data publishing framework, especially pertaining to the issues of persistent identifiers, the data usage index, and a data citation mechanism. This was further discussed during the DataCite Summer Workshop 2010 [70]. Members of the Task Group were engaged in exploring solutions to various components of the data publishing framework, some of which are included in this issue [57,59,61,71], and some published elsewhere [69,72,73] and MJ Costello, WK Michener, *et al.*, personal communication.

In January 2011, the US National Science Foundation (NSF) implemented a policy requiring all NSF grant applicants to submit data management plans as a part of any grant proposal [74]. This policy change seems to represent a very significant fulfillment of our recommendation, though the exact details of its implementation remain as yet unclear.

We believe that timely implementation of these recommendations and suggested solutions or approaches by the GBIF network will support much needed recognition for individual and institutional efforts in management and publishing of primary biodiversity data. GBIF's support of these recommendations should be of critical importance in establishing their credibility and winning their widespread adoption. Implementation of these recommendations should substantially increase the volume of available primary biodiversity data, substantiating public investment in biodiversity science and conservation of biotic resources.

The DPF TG notes several preliminary efforts to implement these recommendations by the GBIF Secretariat. The DPF TG recommendation on incentivizing efforts for metadata authoring has led the GBIF secretariat to commission Pensoft Publishers to create a 'data paper' [71] section in four of its journals (*BioRisks*, *PhytoKeys*, *NeoBiota* and *ZooKeys*) alongside a 'push-button' mechanism to generate XML-encoded manuscripts from metadata descriptions to be submitted directly to the publisher for peer review and editorial evaluation and publication in a form of a data paper [71]. The BIP Index, an exploratory study to develop metrics to determine country-level biodiversity informatics potentials, has been undertaken [61]. GBIF was, moreover, invited to be part of the group of experts convened by the CODATA (the Committee on Data for Science and Technology) to develop an approach to data citation.

We were mandated to make recommendations for potential uptake by the GBIF network. However, we believe that these recommendations apply to the broader biodiversity informatics and ecoinformatics community. Nevertheless, we reiterate that the GBIF network is the most natural venue to kick-start the early implementation of these recommendations. As GBIF enters into its third phase, in which it aspires to be the foremost global resource for biodiversity information [75], an early leadership and proactive step towards implementation of these recommendations is imperative for its success.

## Conclusions and future work

The effective sharing of research data has become a goal of the international research community. Implementation of these recommendations should expedite the progress of archiving, curation, discovery and publishing of primary biodiversity data, because scientists and originators of data will realize the value and incentives for such efforts. We believe that implementation of our recommendations by the GBIF network, and its adoption by similar initiatives such as GEO-BON, IPBES and CBD, will contribute to a much needed global research infrastructure and specifically to an open access regime in biodiversity and conservation science. We further believe that adoption should encourage the evolution of a richly informed virtual research space for future studies in biodiversity [76]. However, we believe that, ultimately, implementation of these recommendations will depend less on policy-political decisions or technical-infrastructural development and primarily on cultural, normative and attitudinal changes by individuals, institutions and organizations.

## Competing interests

The authors declare that they have no competing interests.
